# Structural Insights into Alphavirus Assembly Revealed by the Cryo-EM Structure of Getah Virus

**DOI:** 10.3390/v14020327

**Published:** 2022-02-05

**Authors:** Ming Wang, Zhenzhao Sun, Chenxi Cui, Shida Wang, Decheng Yang, Zhibin Shi, Xinyu Wei, Pengfei Wang, Weiyao Sun, Jing Zhu, Jiaqi Li, Bingchen Du, Zaisi Liu, Lili Wei, Chunguo Liu, Xijun He, Xiangxi Wang, Xinzheng Zhang, Jingfei Wang

**Affiliations:** 1 State Key Laboratory of Veterinary Biotechnology, Harbin Veterinary Research Institute, Chinese Academy of Agricultural Sciences, Harbin 150036, China; wangming@caas.cn (M.W.); sunzhenzhao@nefu.edu.cn (Z.S.); wangshidadjjc@163.com (S.W.); yangdecheng@caas.cn (D.Y.); Szhibin1994@126.com (Z.S.); xinyuwei12@163.com (X.W.); wang_pengfeia@163.com (P.W.); sunweiyao1@126.com (W.S.); zhujingeau@126.com (J.Z.); Lijiaqi0103@126.com (J.L.); bingchendu@126.com (B.D.); liuzaisi@126.com (Z.L.); weilili@caas.cn (L.W.); liuchunguo@sinder.cn (C.L.); hexijun@caas.cn (X.H.); 2 National Laboratory of Biomacromolecules, CAS Center for Excellence in Biomacromolecules, Institute of Biophysics, Chinese Academy of Sciences (CAS), Beijing 100101, China; jeffchenxi@163.com

**Keywords:** alphavirus, Getah virus, cryo-EM, viral assembly, block-based reconstruction

## Abstract

Getah virus (GETV) is a member of the alphavirus genus, and it infects a variety of animal species, including horses, pigs, cattle, and foxes. Human infection with this virus has also been reported. The structure of GETV has not yet been determined. In this study, we report the cryo-EM structure of GETV at a resolution of 3.5 Å. This structure reveals conformational polymorphism of the envelope glycoproteins E1 and E2 at icosahedral 3-fold and quasi-3-fold axes, which is believed to be a necessary organization in forming a curvature surface of virions. In our density map, three extra densities are identified, one of which is believed a “pocket factor”; the other two are located by domain D of E2, and they may maintain the stability of E1/E2 heterodimers. We also identify three N-glycosylations at E1 N141, E2 N200, and E2 N262, which might be associated with receptor binding and membrane fusion. The resolving of the structure of GETV provides new insights into the structure and assembly of alphaviruses and lays a basis for studying the differences of biology and pathogenicity between arthritogenic and encephalitic alphaviruses.

## 1. Introduction

Getah virus (GETV) is a mosquito-borne RNA virus belonging to the Alphavirus genus in the Togaviridae family, which contains several human pathogens, such as chikungunya virus (CHIKV), Semliki Forest virus (SFV), Venezuelan equine encephalitis virus (VEEV), and Sindbis virus (SINV), etc. Alphaviruses have a global distribution and cause outbreaks in human and animals [1,2,3,4]. Based on their historical geographic distribution and the clinical symptoms they cause, alphaviruses are classified into two subgroups: Old World (OW) and New World (NW) alphaviruses [5]. GETV is an OW alphavirus, which typically cause rheumatic disease and are also known as arthritogenic alphaviruses. GETV was first isolated in Malaysia in 1955 from a Culex mosquito and then spread to Japan, China, Korea, and Australia [6,7,8,9,10]. As a mosquito-borne virus, GETV has an extensive host range, which includes pig, fox, cattle, horse, and human [9,11,12,13,14]. It usually causes pyrexia, leg oedema in horses [15], stillbirth in pregnant pigs [13], and neurological symptoms and death in piglets [16], leading to economic losses to the livestock industry and posing a threat to public health. There are no licensed vaccines or effective antiviral therapies for most alphaviruses.

Alphaviruses have a ~12 kb length single-stranded positive-sense RNA genome, which consists of two open reading frames (ORFs), the N-terminal and C-terminal ORFs. The former encodes four nonstructural proteins, nsps 1–4, which contribute to virus replication, host immune escape, and protein modification [4]; the latter encodes four structural proteins, capsid protein (CP), E1, E2, and E3. They serve as the skeletal molecules of the virions and participate in various processes throughout the viral life cycle. The structural proteins are synthesized initially as a long polyprotein, which undergoes post-translational processing and is cleaved into CP, E1, p62, 6K, and transframe (TF). The p62 is further cleaved into E2 and E3 by furin. This cleavage progress is essential for virus maturation [17].

The virions of alphaviruses have a viral core surrounded by an external envelope with a diameter of about 70 nm. The viral core is formed by 240 copies of the CPs and a linear genome RNA molecule. The outer envelope comprises 80 trimeric spikes each containing three copies of heterodimers E1/E2 [18,19] or heterotrimers E1/E2/E3 [20], which are arranged on the surface of the viral particles with a T = 4 icosahedral symmetry. E1 is composed of three major distinct structural domains: a cytoplasmic tail, a transmembrane helix (TM), and an ectodomain, which includes three subdomains of DI, DII, and DIII [21]. Aside from its functions as one of the skeleton components of the viral particles, the most important role E1 plays is to fuse the viral and cellular membranes during virus entry, which is mediated by the fusion loop (FL) located in the DII [22,23]. The structure of E2 is similar to E1. Its ectodomain contains four subdomains: domains A, B, C, and D [21]. E2 is one of the major antigens of alphaviruses, and is also responsible for binding host cell receptors [24]. The diversity of the receptor-binding domain might determine the host tropisms of alphaviruses. The CP provides a physical protection shell for the viral genome RNA and may also be involved in viral genome replication [25].

Structural investigations have been conducted on several alphaviruses, which include CHIKV [21], VEEV [26], eastern equine encephalitis virus (EEEV) [18], Mayaro virus (MAYV) [19], Barmah Forest virus (BFV) [27], western equine encephalitis virus (WEEV) [28], Ross River virus (RRV) [29], and SINV [20]. These studies have disclosed both common and distinct structural features of the above alphaviruses. However, as a large family, alphaviruses show great diversities in host tropism, genetics, pathogenicity, and other biological characteristics, and the structure of GETV has not yet been determined. In this study, we resolve the cryo-EM structure of an arthritogenic alphavirus GETV to a resolution of 3.5 Å. This study provides a structural basis for further exploring the biological differences among alphaviruses.

## 2. Materials and Methods

### 2.1. Production and Purification of GETV Virions

The GETV strain SC483 used in this study was isolated from clinically healthy pigs in Sichuan Province in 2018 by our laboratory. Vero cells (ATCC, CCL-81) were grown in the Dulbecco’s Modified Eagle Medium (DMEM, supplemented with 10% fetal bovine serum and 1% penicillin-streptomycin) for 24 h, and then infected with GETV at a multiplicity of infection (MOI) of 0.1. The supernatant containing mature virions was harvested 24 h post-infection when the viral titer reached 10^7.5^ TCID_50_/mL and then centrifuged to remove cell debris. The supernatant (400 mL) was pelleted through a 20% (*w*/*v*) sucrose cushion at 100,000× *g* for 1 h 30 min in a Beckman rotor (32 Ti) at 4 °C, then the viral pellets were gently resuspended in 1 mL phosphate-buffered saline (PBS, pH 7.4) and loaded onto a 25~45% (*w*/*v*) sucrose discontinuous density gradient, centrifuged at 75,000× *g* for 2 h in a Beckman rotor (41 Ti) at 4 °C. The band corresponding to the viral particles was extracted with a syringe and concentrated by centrifuging in PBS buffer (pH 7.4). The final pellets were gently resuspended in 100 μL PBS buffer (pH 7.4). The purity and integrity of the viral particles were checked using an electron microscopy H-7650 (Hitachi, Tokyo, Japan) operating at a voltage of 80 kV.

### 2.2. Cryo-EM Sample Preparation and Data Collection

For cryo-EM grid preparation, an aliquot of 3.5 μL purified viral solution was applied to a freshly O_2_/Ar glow-discharged Quantifoil Au grid (R1.2/1.3, 200 mesh; Quantifoil Micro Tools) using a Vitrobot Mark IV instrument with a blotting force of -3 and a blotting time of 4 s, and the grid was rapidly dipped into a liquid ethane bath.

To improve the quality and efficiency of data acquisition, we used the beam-image shift method to complete data acquiring [30]. Data collection was performed by a 300 kV Titan Krios microscope (Thermo Fisher Scientific, Hillsboro, OR, USA) equipped with direct electron detector GIF-K2 Summit camera (Gatan, Pleasanton, CA, USA) using SerialEM software [31]. Images were recorded at a magnification of 105 kX in super-resolution mode, yielding a 2× binning pixel size of 1.36 Å. Each exposure of 5.5 s was dose fractionated into 32 movie frames leading to a total dose of 30 e-/Å2 and a corresponding dose rate of 10 e/pixel/s. Movies were collected with the defocus values determined from −0.8 μm to −2.5 μm by the CTFFIND4 [32].

### 2.3. Single Particle Reconstruction

Motion correction was performed using the MotionCor2 program [33]. The contrast transfer function (CTF) value of each frame was estimated using CTFFIND4 [32]. A total of 36,066 particles were extracted from 2555 micrographs and subjected to nonreference 2D classification in RELION [34] to filter out particles that failed to generate good average projections. The remaining 30,996 GETV particles were used for 3D reconstruction. In the first-round iteration of 3D autorefinement, reconstruction led to a resolution of 6.0 Å assuming icosahedral symmetry. Then, a mask was applied in the reconstruction to further improve the overall resolution and obtained a virion structure at a resolution of 4.6 Å. The final reconstruction was done by using the block-based reconstruction method [35]. Three blocks were defined (Block 1 was the icosahedral five-fold of type-2 quasi-three-folds, Block 2 was four type-2 quasi-three-folds flanked by two type-1 symmetrical trimers across the icosahedral two-fold axis, and Block 3 was one type-1 symmetrical trimer surrounded by three type-2 quasi-three-folds about the icosahedral three-fold axis) and refined separately to compensate for the flexibility of the structure. The final resolution of each block was determined by gold standard FSC (0.143) in RELION [34].

### 2.4. Model Building and Refinement

The homology models of E1, E2, and CP of GETV were built using a SWISS MODEL server (https://swissmodel.expasy.org, accessed on 5 July 2021). An asymmetric unit of GETV was then assembled using that of CHIKV (PDB: 6NK6) as a reference. The asymmetric unit of GETV was then fit into the reconstruction using Chimera’s Fit in Map tool [36]. The atomic model coordinates were manually corrected and checked using COOT [37] and subsequently optimized with the tool real_space_refinement in the PHENIX software [38] running under the conditions of minimization_global, rigid_body, local_grid_search, secondary structure restraints, morphing, and simulated_annealing. The refinement statistics of the structural model are listed in Table S1. All maps and models were visualized using Chimera [36].

### 2.5. Prediction of Post-Translational Modifications

The amino acid sequences of E1 and E2 of GETV were submitted to the online servers for predicting possible post-translational modifications. N-glycosylation and O-glycosylation sites were predicted using the online servers of NetNGlyc 1.0 (http://www.cbs.dtu.dk/services/NetNGlyc, accessed on 2 August 2021) and NetOGlyc 4.0 (http://www.cbs.dtu.dk/services/NetOGlyc, accessed on 2 August 2021), respectively.

### 2.6. Sequence Alignment

The amino acid sequences of E1, E2, and CP from 12 representative alphaviruses were downloaded from the GenBank database (https://www.ncbi.nlm.nih.gov/genbank/, accessed on 6 August 2021). The sequences were aligned using the Clustal W method implemented in MEGA6. The aligned sequences were displayed by the ESPript 3.0 server [39]. The accession number of these sequences are as follows: GETV (MN478486), CHIKV (KY680355.1), SAGV (AF339483.1), RRV (K00046.1), MAYV (MK070491.1), MIDV (AF339486.1), ONNV (AF079456.1), SFV (NC_003215.1), SINV (MH229928.1), EEEV (AMT80016.1), VEEV (AY741139.1), and WEEV (J03854.1).

## 3. Results

### 3.1. Overall Cryo-EM Structure of GETV Virions

**Figure 1:**
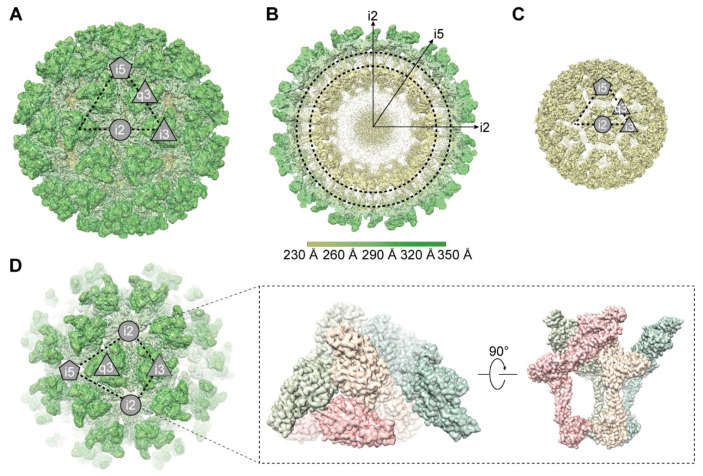
Cryo-EM reconstruction of GETV particles. (**A**) Radially colored 3D reconstruction of GETV particles. The 5-fold (i5) axis and 2-fold (i2) axis are indicated with a pentagon and a circle, respectively. The icosahedral 3-fold (i3) axis and quasi-3-fold (q3) axis are indicated with black triangles. (**B**) The central slice of the GETV virion structure. The lipid bilayer is marked with black dashed circles. (**C**) The 3D reconstruction of inner capsid shell. (**D**) Each asymmetric unit (ASU) is composed of four E1/E2 heterodimers. Left panel, one of the ASU is indicated with a black dashed quadrilateral. Right panel, a top view (left) and a side view (right) of one ASU. Each E1/E2/CP unit within an ASU is distinguished by different colors (pink, wheat, green, and blue).

The GETV (SC483) was isolated from clinically healthy pigs in Sichuan Province in 2018 by our laboratory and was maintained in Vero cells. The supernatant containing mature virions was harvested and purified by ultracentrifugation. To obtain a high-resolution structure of GETV virions, we first applied icosahedral symmetry to the reconstruction. A total of 30,996 viral particles were involved in this process, and obtained the structure at a global resolution of 4.6 Å (Figure S1A,B,D, and Table S1). To overcome the heterogeneity of the particles and further improve the resolution, we then used the block-based reconstruction method to further refine the structure [35]. The three blocks of the T = 4 icosahedral lattice were as follows: Block 1 as the icosahedral five-fold of type-2 quasi-three-folds (B1), Block 2 as four type-2 quasi-three-folds flanked by two type-1 symmetrical trimers across the icosahedral two-fold axis (B2), and Block 3 was one type-1 symmetrical trimer surrounded by three type-2 quasi-three-folds about the icosahedral three-fold axis (B3) (Figure S1C). Each block was refined and reconstructed separately, and the final resolutions of the three blocks were assessed by the gold standard Fourier shell correlations (FSC, 0.143) to be 3.5 Å, 3.7 Å, and 3.7 Å, respectively (Figure S1D,E). The three-dimensional reconstruction clearly showed the organization of dual nested shells of GETV virions, an outer glycoprotein shell, and an inner nucleocapsid shell (Figure 1A–C). Like other alphaviruses, the diameter of the integrated GETV particle is about 700 Å, and that of the nucleocapsid is about 410 Å. The outer glycoprotein shell is constructed by 80 trimers, each of which is composed of three copies of E1 and E2 heterodimers (Figure 1A). The inner nucleocapsid shell is composed of 240 copies of CPs (Figure 1C). Both glycoproteins and CPs are arranged following T = 4 symmetry in forming two icosahedron shells. Each asymmetric unit (ASU) of the virion contains four E1/E2 heterodimers and four CPs (Figure 1D). A slice through the 3D density map (Figure 1B) shows the outer glycoprotein shell and inner nucleocapsid shell is connected by the interaction between CPs and cytoplasmic tails of E1 and E2. In the density map of GETV virions, we did not find the densities that correspond to E3 in SINV [20] and CHIKV [21].

### 3.2. The Structure of Envelope Glycoproteins

**Figure 2:**
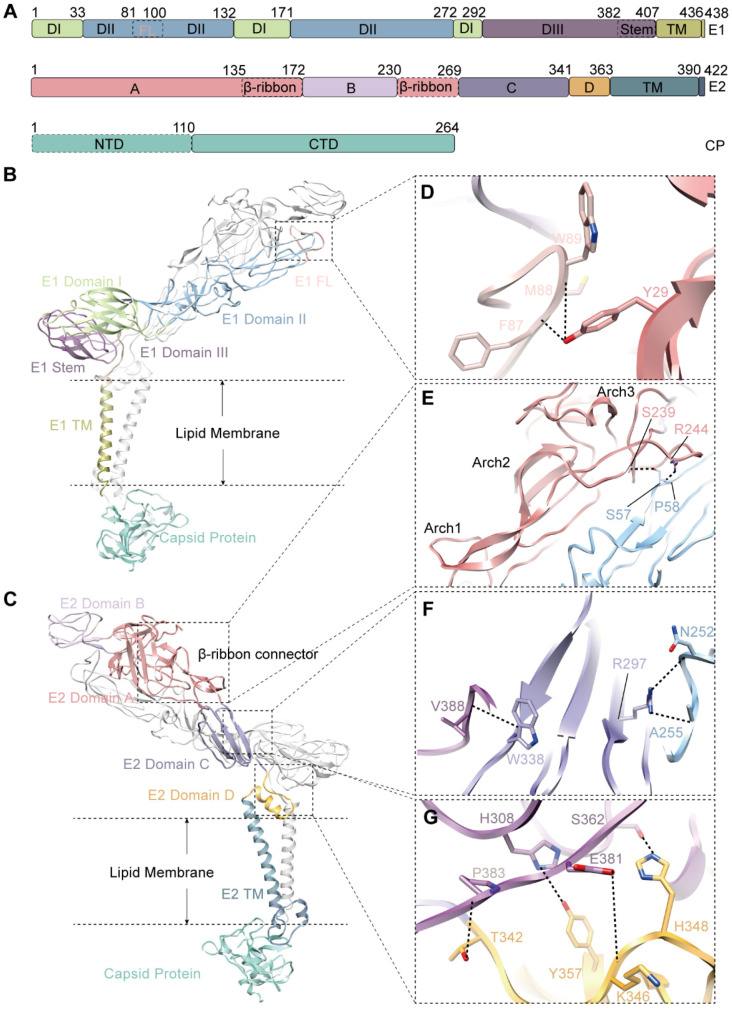
Structure of E1/E2 heterodimer. (**A**) Linear diagrams show the subdomain distributions in E1, E2, and CP with different colors (DI, light green; DII, light blue; DIII, purple; FL, peach puff; TM of E1, olive; domain A, Indian red; domain B, orchid; domain C, medium purple; domain D, orange; TM of E2, slate blue. CP, turquoise). (**B**,**C**) The atomic models of GETV heterodimer displayed with edged ribbon. Subdomains of E1 and E2 are color coded in the same way as (**A**). (**D**–**G**) The primary hydrogen bonds formed between E1 and E2 are labeled with dashed lines.

The glycoprotein E1 is a class II fusion protein. It mediates the fusion of viral and cellular membranes after entry [40,41]. Taking the E1 structure of CHIKV as a reference, we defined the structural domains of E1, which include an ectodomain, a transmembrane helix, and a cytoplasmic tail. The ectodomain of E1 was further divided into three subdomains: domain I (DI, residues 1–33, residues 133–171 and 273–292), domain II (DII, residues 34–132, 172–272), and domain III (DIII, residues 293–407) (Figure 2A,B). The hydrophobic fusion loop (FL, residues 82–100) forms a small hairpin, located at the distal tip of DII domain with a highly conserved Trp89 protruding at the turn (Figure 2D and Figure S2A). FL makes extensive contact with E2 domain A and domain B. The side chain of E2 Y29 from Domain A hydrogen-bond the E1 F87 and E1 M88 from the fusion loop (Figure 2D, Table S2), which may maintain the stability of FL.

The E2 is an immunoglobulin-like glycoprotein and is responsible for the receptor engagement. The domain division of E2 is similar to that of E1. The ectodomain of E2 folds into four distinct subdomains: domain A (residues 1–172, residues 231–269), domain B (residues 173–230), domain C (residues 270–341), and domain D (residues 342–363) (Figure 2A,C). domain B is located at the tip of E2, contains mainly flexible loops, and plays a key role in the receptor recognition. domain A is the center core of the ectodomain of E2. A major component of domain A is the β-ribbon, which connects domain B with its membrane-distal end, and domain C with its membrane-proximate end (Figure 2C). It is composed of an antiparallel β sheet and three arched loops (Figure 2E, Arch1–3). The long, flexible β-ribbon is stabilized by its extensive interactions with DII of E1 (Table S2). At least 14 amino acids of β-ribbon are involved in these interactions, forming three hydrogen bonds between E2 R138 and E1 D253, E2 S239 and E1 S57, and E2 R244 and E1 P58 (Figure 2E, Table S2). domain C is β-sheet riched and facilitates strong interactions with the DII and DIII of E1 by forming several hydrogen bonds as shown in Figure 2F and Table S2. Domain D is membrane-proximal and is important for viral budding and fusing [42]. It interacts with the DIII of E1 by forming several hydrogen bonds (Figure 2G, Table S2).

### 3.3. Interactions among Envelope Glycoproteins in Viral Assembly

**Figure 3:**
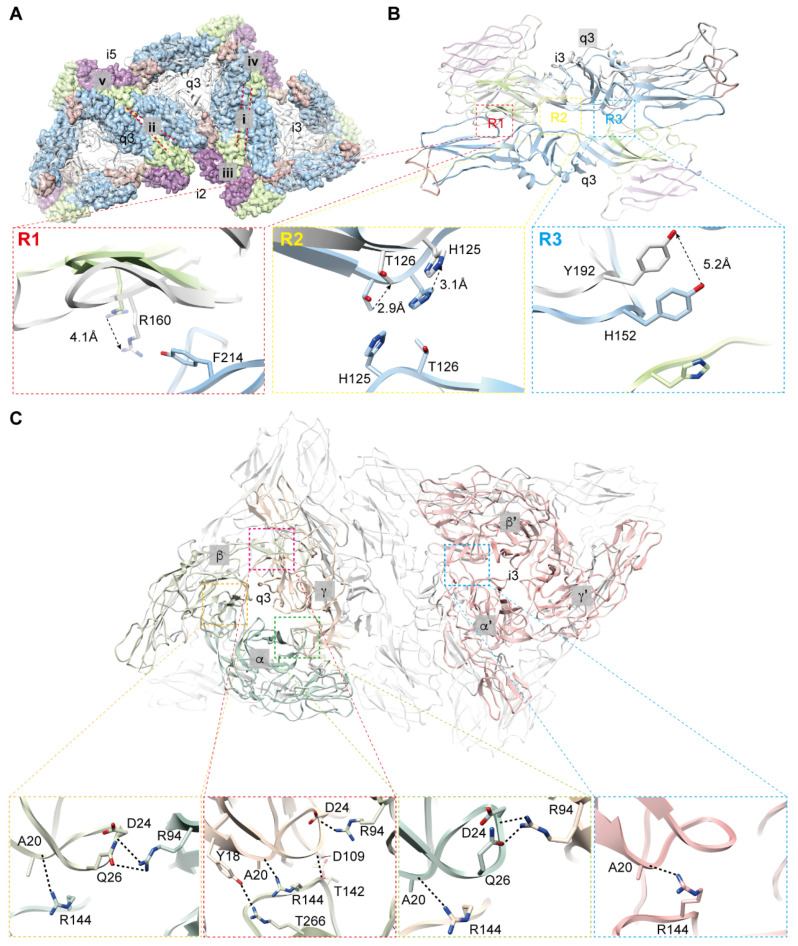
Assembly of envelope glycoproteins. (**A**,**B**) Five types of contacts between two adjacent E1. (**A**) Locations of the five contacts are labeled on the top view of three heterotrimers, indicated by i, ii, iii, iv, and v, respectively. The red dashed lines represent the interacting edges between two adjacent E1. Subdomains of E1 are color coded in the same way as Figure 2A. (**B**) Conformational changes between E1 from i3 and q3. We overlapped the two E1 from q3, and structural differences between the E1 from i3 (colored) and q3 (grey) are observed. Three contacts (R1, R2, and R3) are enlarged, showing the movement (indicated with dashed arrows) of the major residues. (**C**) Contacts between E2 from i3 and q3. Top view of E2 in the heterotrimers. E2 from q3 are labeled α (light blue), β (light green), and γ (wheat), and from the corresponding position of i3 are labeled α’ (pink), β’ (pink), and γ’ (pink), respectively. Interactions of α/β, β/γ, α/γ, and α’/β’ (β’/γ’ and α’/γ’ are identical with α’/β’) are enlarged. Dashed lines indicated hydrogen bonds.

There are five types of interactions between two neighboring E1 on the virion. These interactions are formed between icosahedral 3-fold (i3) axis and quasi-3-fold (q3) axis, two neighboring q3, 2-fold (i2) axis and i3, i2 and q3, and near icosahedral 5-fold (i5) axis; the interactions are designated as types i, ii, iii, iv, and v, respectively (Figure 3A, Table S3). Type i and type ii correspond to the previously described Type I and Type II interactions, respectively [20]. Unlike the constant interaction between the two E1 in type i, the distance between the two E1 in type ii is closer near the i5 axis but is separated away from i5 (Figure 3A). To find the conformational changes between type i and type ii interactions, we overlapped the two E1 from q3 and observed the structural differences between the E1 from i3 and q3. Three contact regions (R1, R2, and R3) between the two E1 were found in the type i interaction, but only one (R1) was found in the type ii interaction (Figure 3B). We then compared the conformational differences of the primary amino acids in these three regions of the two type interactions. In R1, the distances between residues in E1 of type ii are closer than that of type i, as indicated by the displacement of R160 (Figure 3B, R1), leading to a more extensive interface that may compensate for the missing interactions in R2 and R3. The absent interactions in type ii result from the upward movement of involved residues as shown in Figure 3B (R2 and R3), the residues T126 and H125 in R2 and Y192 in R3 do not make contact with their counterparts. These differences in the interaction between two adjacent E1s may contribute to the maintenance of the spherical surface of viral particles and play essential roles in viral particle assembly.

The E2 takes different conformations in forming the trimers at i3 and q3 (Figure 3C). The E2 in i3 follows an icosahedral 3-fold symmetry, and residues involved in the interfaces between two adjacent E2 are identical. However, the E2 in q3 are arranged with a quasi-3-fold symmetry, leading to different amino acids being involved in the three interfaces (Table S4). To distinguish the E2 from different locations, we labeled the E2 as α, β, and γ in q3 and α’, β’, and γ’ in i3 (Figure 3C). The interaction between two adjacent E2 is mainly performed by the contacts of domain A and β-ribbon. The residues involved in these contacts include Y18, A20, D24, Q26, R94, and D109 from domain A and T142, R144, and T266 from β-ribbon. They form a hydrogen bond network to stabilize the contact of these two domains (Figure 3C). Interestingly, an obvious difference was found in the numbers of hydrogen bonds between adjacent E2 in i3 and q3. There is only one putative hydrogen bond between A20 from domain A and R144 from β-ribbon in E2 of i3. However, at least three hydrogen bonds are possibly formed between domain A and β-ribbon in E2 from q3 (Figure 3C). The increased hydrogen bonds between the contacts of E2 from q3 are probably to overcome the forces produced by the conformational changes of E2 in assembling quasi-3-fold symmetry, while in assembling a real 3-fold symmetry in i3, less force is needed to form a stable contact between two adjacent E2s.

### 3.4. Structure and Assembly of Capsid Protein

**Figure 4:**
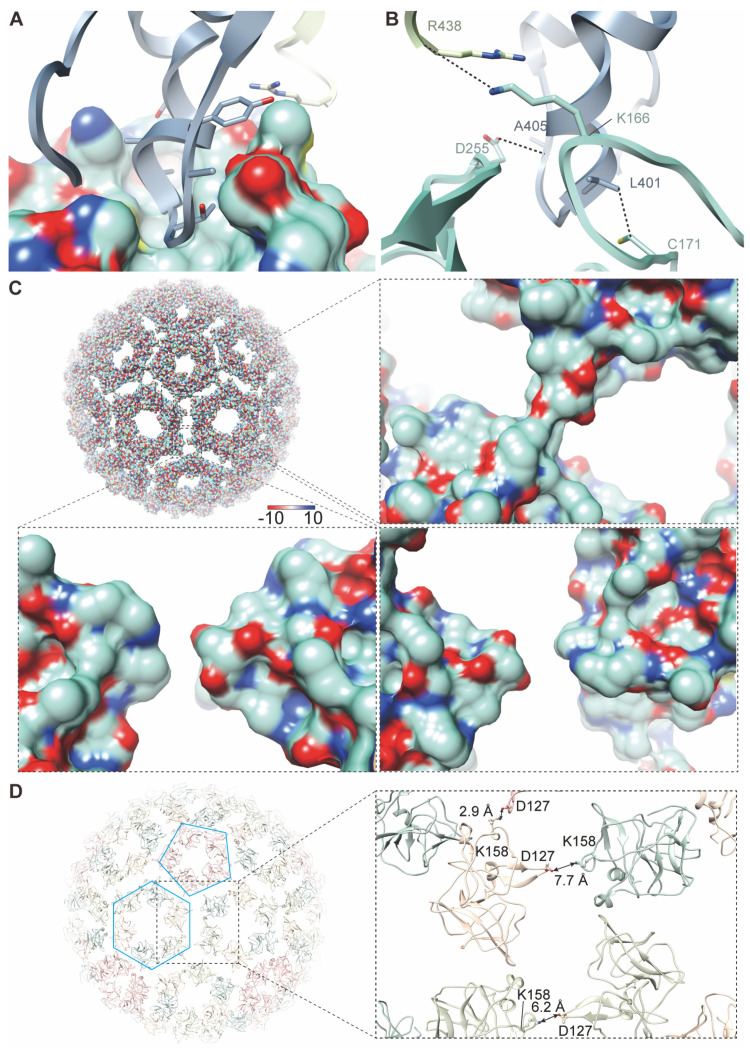
Structure and assembly of CP. (**A**) Interaction between CP and E2 is mediated by the cytoplasmic tail of E2 inserted into a hydrophobic pocket on the surface of CP. (**B**) The main residues involved in the interaction between CP and the cytoplasmic tail of glycoproteins (E1 and E2). The dashed lines represent hydrogen bonds. (**C**) Surface potential of CP. The enlarged images show that the electrostatic interaction between a pentamer and a hexamer is mediated by the complementary charged amino acids. The electrostatic potential ranges from negative (red) to positive (blue). (**D**) The overall atomic model of capsid. The four copies of CPs in each of the ASUs is colored light blue, light green, wheat, and pink, respectively. A pentamer and a hexamer are labeled with a pentagon and a hexagon, respectively. The enlarged image shows the interactions between CPs. The distances between D127 from pentamer/ hexamer and K158 from hexamer are labeled.

CP is a chymotrypsin-like protein. The N-terminal domain (NTD, residues 1–110) of CP is disordered in our density map, while the structure of the C-terminal domain (CTD, residues 111–264) is well defined in this map. There is a hydrophobic pocket on the upper surface of CTD (Figure 4A). The cytoplasmic tail of E2 engages with this pocket and forms a stable connection between CP and E2 through hydrophobic forces, and this engagement is further enhanced by three additional hydrogen bonds (CP C171 and E2 L401; CP D255 and E2 A405; CP K166 and E1 R438) (Figure 4B).

Similar to other alphaviruses, a total of 240 CPs are assembled into 30 hexamers and 12 pentamers. The interface between CPs within a pentamer is different from that within a hexamer, but both are mediated by electrostatic interactions (Figure 4C). The interaction between a pentamer and a hexamer is confirmed by the close contacted residues D127 and K158, which have complementary charges (Figure 4D). However, the interaction between two hexamers, which was reported in the structure of MAYV [19], is absent from our density map of GETV (Figure 4C), which is indicated by the larger distance between the two conserved residues D/E 127 and K/R 158 (>6Å) (Figure 4D and Figure S2C).

### 3.5. Hydrophobic Pocket and Newly Discovered Densities in GETV

**Figure 5:**
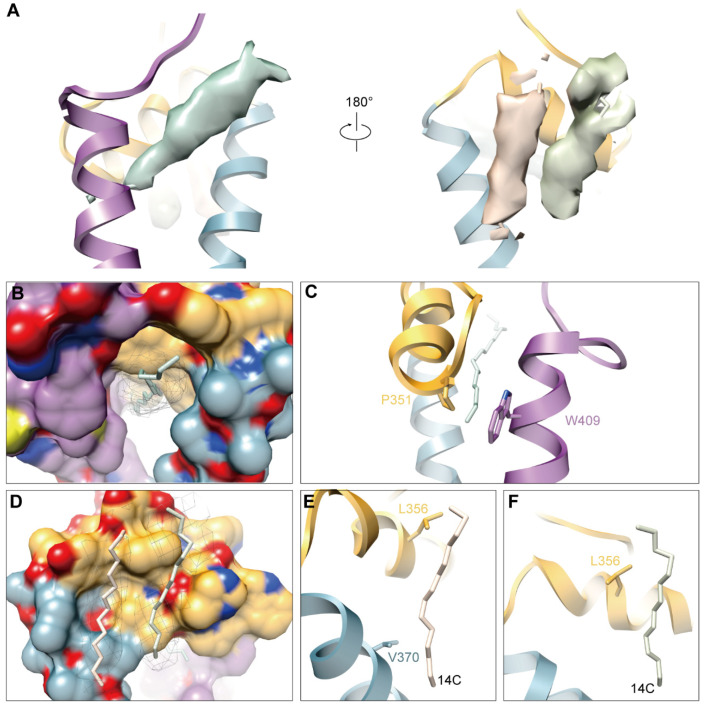
Hydrophobic pocket and three extra densities. (**A**) Three extra densities located by the E1/E2 transmembrane helix. The density responses to the previously identified “pocket factor” is showed as light green surface with an 18C model fitted in. The two newly identified extra densities are presented as wheat and olive surfaces with two 14C models fitted in, respectively. (**B**) The hydrophobic pocket with an 18C molecule modelled in. (**C**) A “lid-like” structure formed by P351 from E2 and W409 from E1 at the posterior of the pocket. (**D**) Two putative fatty acids are fitted in the positions of the two extra densities, showing they attach to two hydrophobic grooves on the side surface of domain D of E2 and transmembrane helix (TM), respectively. The two putative fatty acid with 14C are displayed as stick. (**E**,**F**) Major amino acids from E2 participated in the interaction with the two densities.

As observed in other alphaviruses, there is also a hydrophobic pocket between transmembrane helices of E1 and E2 in GETV, and there is a long extra density in the upper part of the pocket (Figure 5A,B), which has also been found in other alphaviruses [19,20,26] This extra density was presumed to be an 18C fatty acid and named “pocket factor” by the authors reporting the cryo-EM structure of SINV [20]. The majority of the residues composing the pocket are hydrophobic amino acids, which form hydrophobic contact with the pocket factor. Most of them are largely conserved among alphaviruses (Figure S2A,B). In the posterior of the pocket, there are two residues (E2 P351 and E1 W409) forming a lid-like structure, which may prevent the release of the pocket factor (Figure 5C). Strikingly, we found two additional densities by the hydrophobic pocket (Figure 5A,D) that have not been found in other structure of alphaviruses. We modeled two fatty acids with 14C. They were then fitted into these densities and were found to match well with them (Figure 5A,D). Therefore, we presumed that these two densities are probably 14C fatty acids and participate in the stability of E1/E2 heterodimers. The presumed 14C densities are stabilized by interacting with L356 and V370 of E2 (Figure 5E,F). The components and functions of these two molecules should be studied in the future.

### 3.6. N-glycosylations in E1 and E2

**Figure 6:**
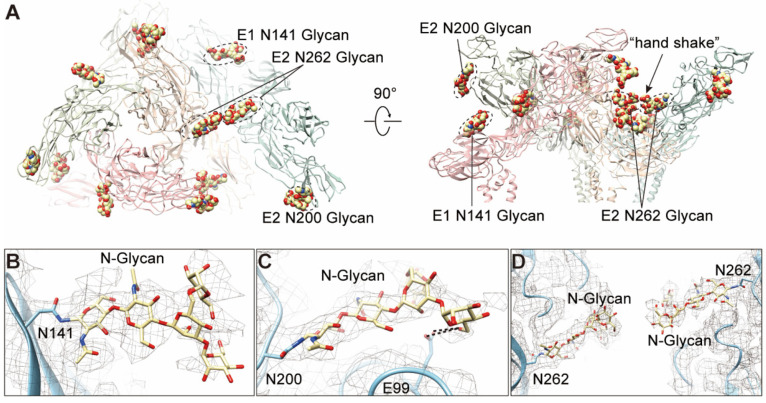
The N-linked glycans in E1 and E2. (**A**) Top view and side view of an asymmetric unit (ASU) showing the locations of the N-linked glycans. The E1/E2 heterodimers are colored blue, green, wheat, and pink, respectively. (**B**–**D**) Local density and structure of the three N-linked glycans. The glycan linked on N141 from E1 is shown in (**B**); The glycan linked on N200 from E2 has a close contact with E99 by forming a hydrogen bond (**C**); The glycans linked on N262 from two adjacent E2 form a “hand shake”-like structure (**D**).

Glycosylation is one of the post-translational modifications widely found in eukaryotes and viruses. To investigate whether this modification occurs in E1 and E2 of GETV, we predicted the N-glycosylation of E1 and E2 proteins using the NetNGlyc 1.0 server. Results showed that there are three predicted N-glycosylation sites, one of which is located in E1 (^141^NQT^143^) and the other two are located in E2 (^200^NCT^202^ and ^262^NST^264^). We observed the densities of glycan chains at the predicted sites in the cryo-EM density (Figure 6A–D), and high mannose was added to the densities when modeling the N-glycans. Further, we predicted the O-glycosylation using the NetOGlyc4.0 server and obtained several predicted O-glycosylation sites; however, the densities of these glycan chains are absent in our cryo-EM density map.

The ^141^NXT^143^ (X is any amino acid except Proline) motif in E1 is highly conserved in arthritogenic alphaviruses (Figure S2A); however, similar motifs were found at N139 in SINV and WEEV, a more distant N134 site in EEEV and VEEV, agree with the previous study that 141 glycosylation in E1 protein are structurally conserved among alphaviruses [19]. The N141 is located at the tip of the β-hairpin of DI (Figure 6A,B). This glycosylation was presumably associated with virus-induced disease [43], and the function mediated by the N141 glycosylation seemed conserved throughout alphaviruses.

The ^200^NXT^202^ motif in E2 was found in SFV, MIDV, SAGV, and RRV but absent in MAYV, ONNV, CHIKV, and other encephalitic alphaviruses (Figure S2B). The N200 is located at the turn of the β-hairpin of domain B (Figure 6A,C). Interestingly, we observed two pairs of possible hydrogen bonds formed between O6 of the glycan and E99 in E1, respectively (Figure 6C). The E99 is located at the terminal of FL and is highly conserved among alphaviruses (Figure 6C and Figure S2A), which seems to stabilize the FL. In addition, we also observed several residues (C63, C96, and E99 in E1; T202 in E2) involved in the interactions with this glycan (Table S5).

The ^262^NXT^264^ motif is conserved among all the arthritogenic alphaviruses but not in encephalitic alphaviruses (Figure S2B). This glycan between two adjacent spikes forms a “hand shake” structure (Figure 6A,D), which was also found in MAYV [19]. Given that the site locates near the receptor-binding domain of arthritogenic alphaviruses, it might participate in the engagement of receptors.

## 4. Discussion

GETVs have been reported to cause fever in humans and have resulted in severe animal disease outbreaks in China [9,11,12,13,14], indicating that GETVs pose an increasing threat to both animal productions and public health. Given the structure of GETV has not yet been determined, we launched the current study and resolved the cryo-EM structure of GETV to 3.5 Å resolution, based on which the composition and assembly of GETV virions have been explored extensively and revealed different contacts between E1/E1, E2/E2 from i3 and q3. Additionally, we identified the extra densities existed near domain D of E2 and three N-glycans (E1 N141, E2 N200, and E2 N262). These structural characteristics provide references for exploring the differences of biology and pathogenicity between arthritogenic and encephalitic alphaviruses.

The E1 and E2 involved in the assembly of spikes of alphaviruses show conformational polymorphism. While assembling into an icosahedral particle, the spikes form two types of 3-fold axis in alphaviruses, the i3 axis and q3 axis, leading to differences in conformation and interaction of spike proteins in these two locations. This conformational change results in five types of interactions between E1 in GETV. We compared the interactions between two E1 from q3/q3 and q3/i3 and found a gradually increased distance between two E1 at q3/q3 interfaces away from the i5 axis, which was also reported in SINV [20]. These conformational variations of E1 at the interface of q3/q3 lead to one enhanced contact (R1) and two lost contacts (R2 and R3) compared to the E1 at the interface of q3/i3. The assembly of E2 at the positions of i3 and q3 shows a clear difference indicated by an increase in the number of hydrogen bonds between two adjacent E2s. The conformational polymorphism of E1 and E2 at the positions of i3 and q3 is believed to be a necessary organization in forming a curvature surface during the assembly of virions.

The glycosylation of viral proteins has been extensively studied, showing that this kind of modification is involved in multiple functions or processes of the viral life cycle, such as recognition of host receptors, immune escape, and virulence and pathogenicity [44,45]. We analyzed the glycosylation in the glycoproteins of E1 and E2 in GETV and identified one N-glycosylation from E1 (N141) and two from E2 (N200 and N262). The N-glycans at similar sites were also reported in several other alphaviruses, including CHIKV [21,46], MAYV [19], SINV [20,47], RRV [43], and VEEV [26]. The glycosylation at N141 of E1 was proven to be related to the virulence of RRV [43] and SINV [47]. The E2 N200-linked glycan seemed to stabilize the FL of E1 by forming hydrogen bond, and may be involved in the pH-dependent conformational changes of E1 and membrane fusion. The E2 N262-linked glycan was highly conserved in arthritogenic alphaviruses, and might be associated with the receptor binding and pathogenicity of alphaviruses [48]. The functions of these glycosylations in GETV are currently unknown and should be further explored.

We mapped currently known epitopes of alphaviruses on the structure of GETV and compared the differences of residues composing the epitopes between two types of alphaviruses. This mapping showed that the residues involved in the epitopes in E1 are relatively conserved among all the alphaviruses; however, distinct differences of amino acids at the antigenic sites of E2 were observed between arthritogenic and encephalitic alphaviruses, indicating that consensus subunit vaccines against all alphaviruses can possibly be developed based on E1. The variations of the residues on the epitopes in E2 might be also associated with pathogenicity differences between the two types of alphaviruses.

The resolution of the GETV is moderate (3.5 Å), and some atomic details of the structural proteins cannot be clearly defined. Given the polymorphism of alphaviruses, a structure with higher resolution might be achieved either by increasing the number of viral particles or by adopting an optimized virus purification strategy [49].

In this study, we have presented the cryo-EM structure of GETV virions with a near-atomic resolution. The results provide insights into the organization and assembly of another member of the alphavirus family. The structure provides new insights into the structure and assembly of alphaviruses and lays a basis for studying the differences of biology and pathogenicity between arthritogenic and encephalitic alphaviruses.

## Data Availability

The cryo-EM density map of envelope glycoprotein of GETV; Block 1 (envelope glycoprotein), Block 2, and Block 3; Block 1 (capsid); and capsid core have been deposited in the Electron Microscopy Data Bank under accession codes EMD-32412, EMD-32415, EMD-32414, EMD-32413, EMD-32426, and EMD-31966, respectively. The atomic coordinate of envelope glycoprotein, Block 1 (capsid), and capsid core has been deposited in the Protein Data Bank under accession codes 7WC2, 7WCO, and 7VGA, respectively.

## Supplementary Materials

The following supporting information can be downloaded at: https://www.mdpi.com/article/10.3390/v14020327/s1, Figure S1: cryo-EM reconstruction of GETV virions; Figure S2: multiple sequence alignment of the structural proteins from 12 alphaviruses; Table S1: cryo-EM data collection and processing; Table S2: amino acids involved in E2/E1 interface; Table S3: amino acids involved in E1/E1 interface; Table S4: amino acids involved in E2/E2 interface; Table S5: glycans interaction with E1 and E2.
