# Supplementary material for: Structural Insights into Alphavirus Assembly Revealed by the Cryo-EM Structure of Getah Virus
Source: Viruses. 2022 Feb 5;14(2):327. doi: 10.3390/v14020327 (PMC8876998; doi:10.3390/v14020327)
Supplement: Supplementary file 1 [file viruses-14-00327-s001.zip › Supplementary/Suplementary figures.pdf]

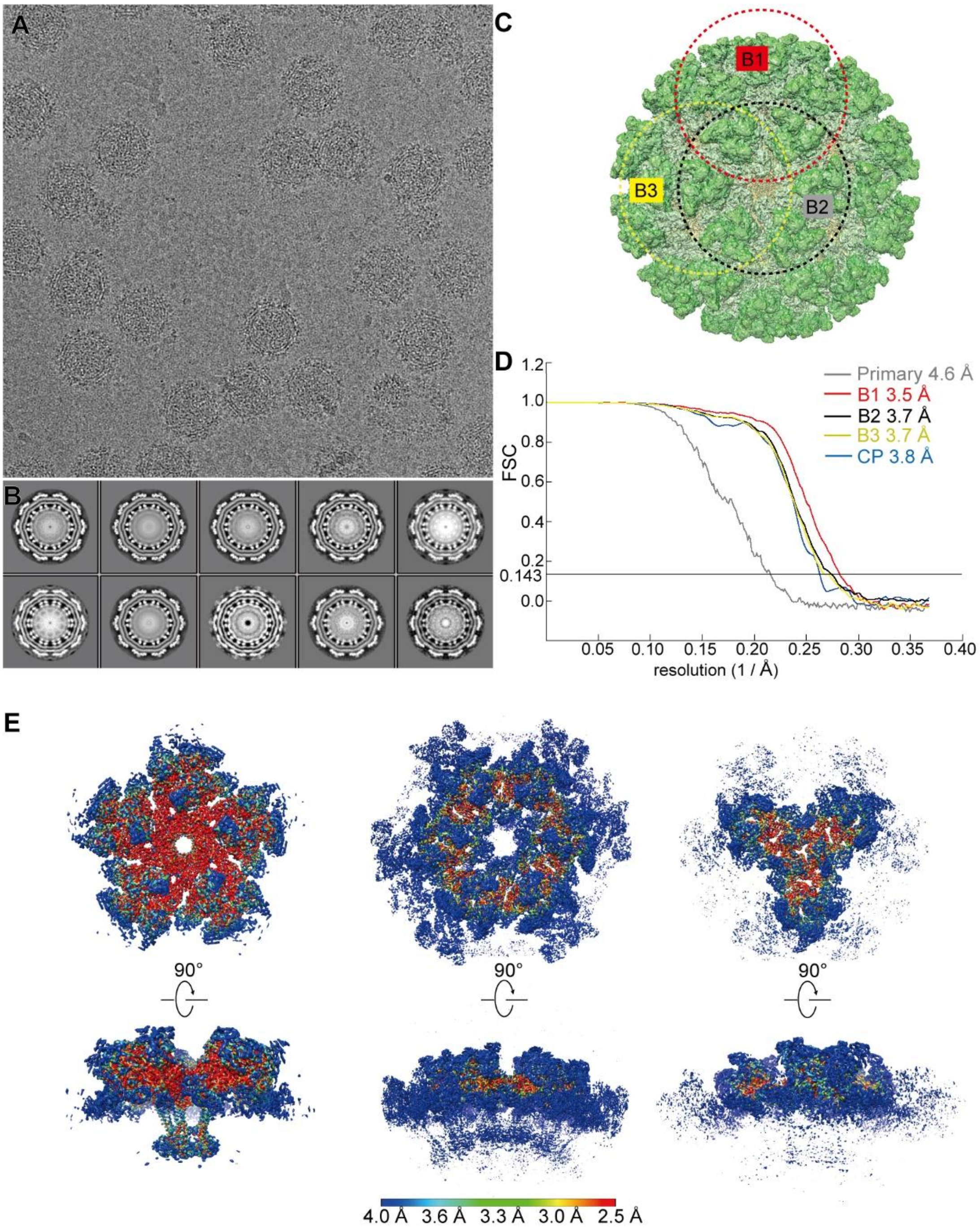

Figure S1. Cryo-EM reconstruction of GETV virions. (**A**) Cryo-EM micrographs of purified GETV virions. (**B**) Two-dimensional classification scheme of GETV virions. (**C**) Location of the three blocks (B1-3) selected for conducting the Block-based reconstruction. B1, B2, and B3 are indicated by a red, black, and yellow dashed circle, respectively. (**D**) Fourier Shell Correlation (FSC 0.143) profile of GETV Cryo-EM density map. The primary resolution curve is colored grey, and the final resolution curves of three blocks and CP are colored red, black, yellow, and blue, respectively. (**E**) Local resolution of the three blocks B1 (left), B2 (middle), and B3 (right).

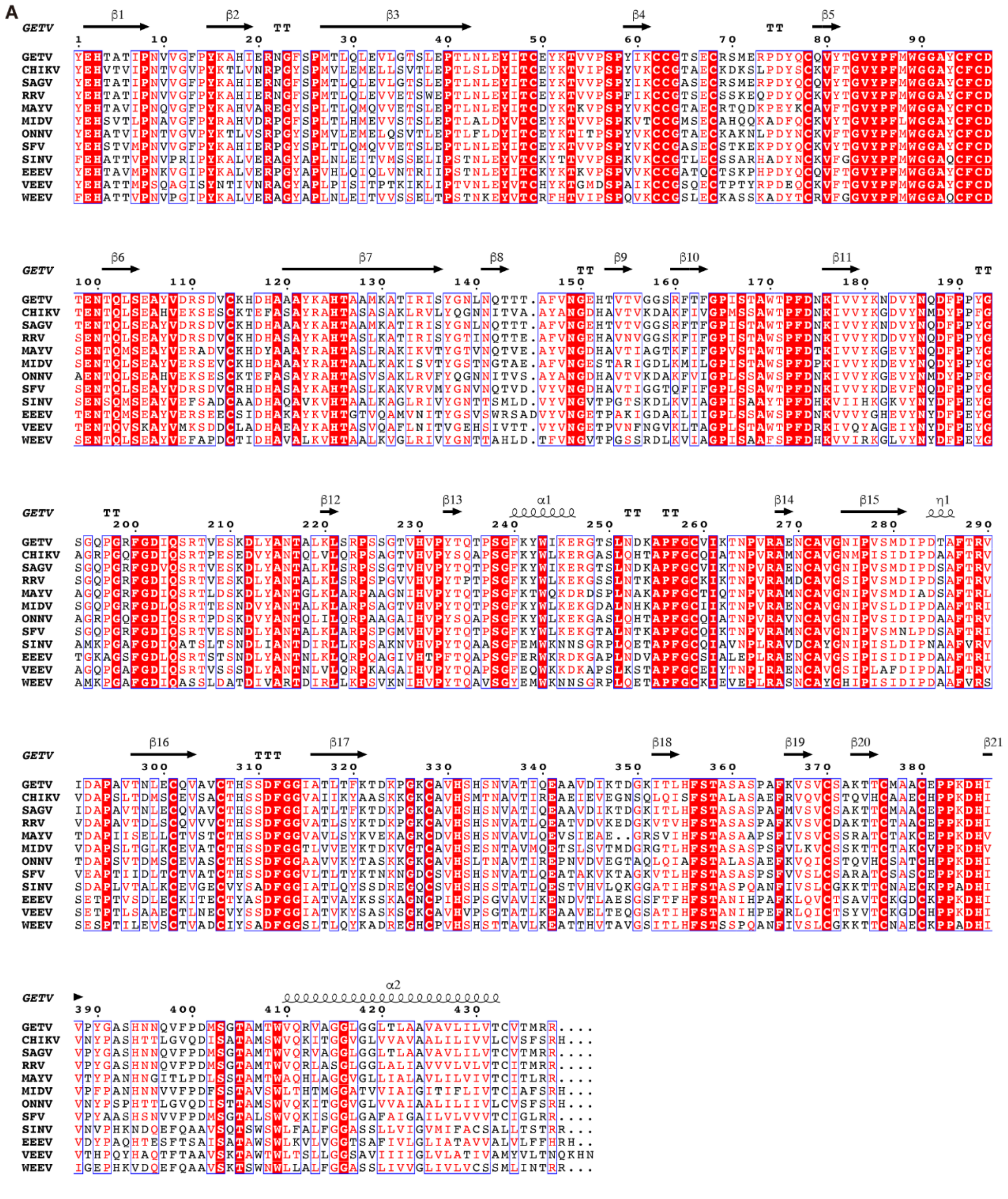
A
GETV
β1 β2 β3 β4 β5 β6 β7 β8 β9 β10 β11 β12 β13 α1 β14 β15 η1 β16 β17 β18 β19 β20 β21 α2
GETV
CHIKV
SAGV
RRV
MAYV
MIDV
ONNV
SFV
SINV
EEEV
VEEV
WEEV

B

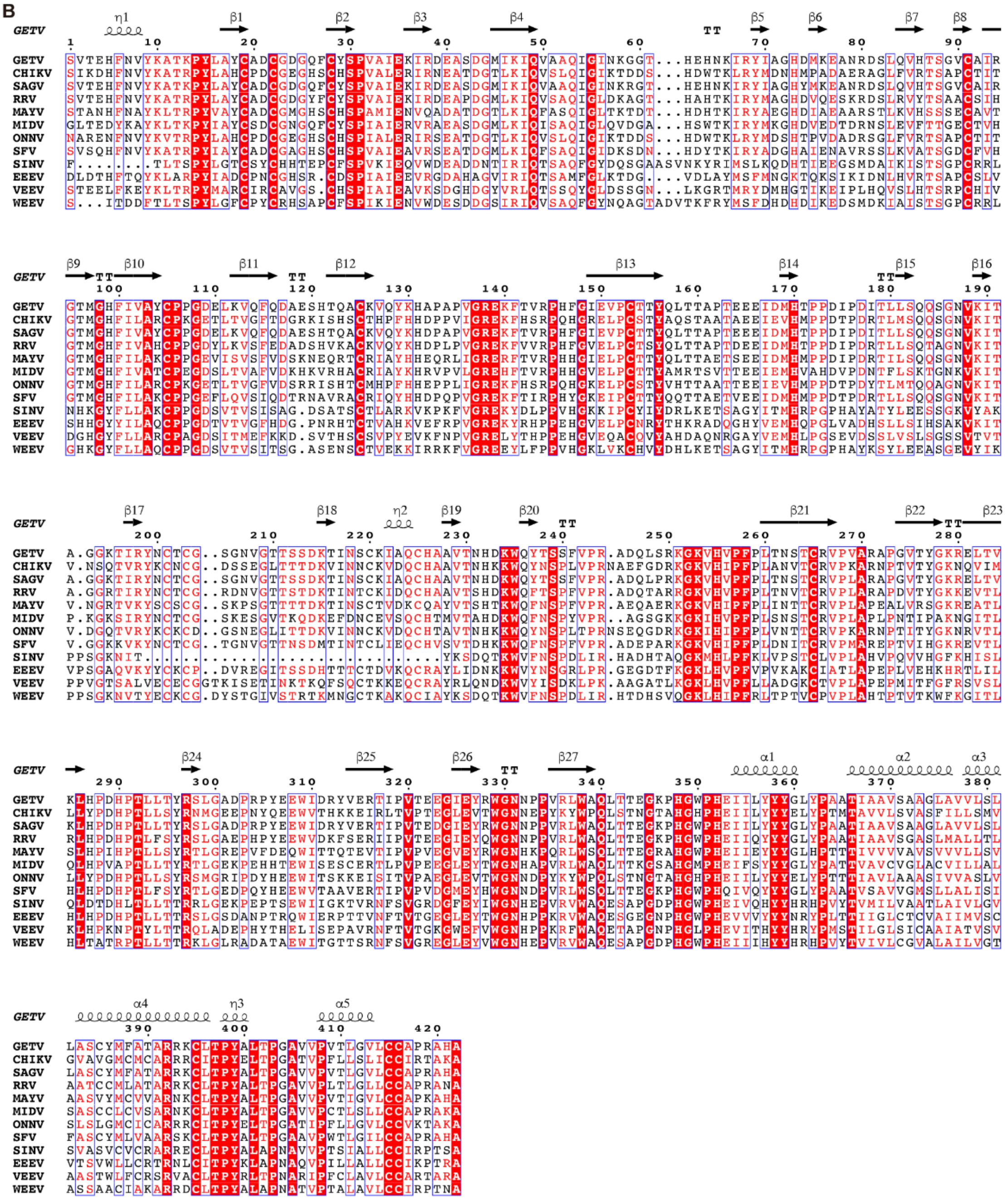

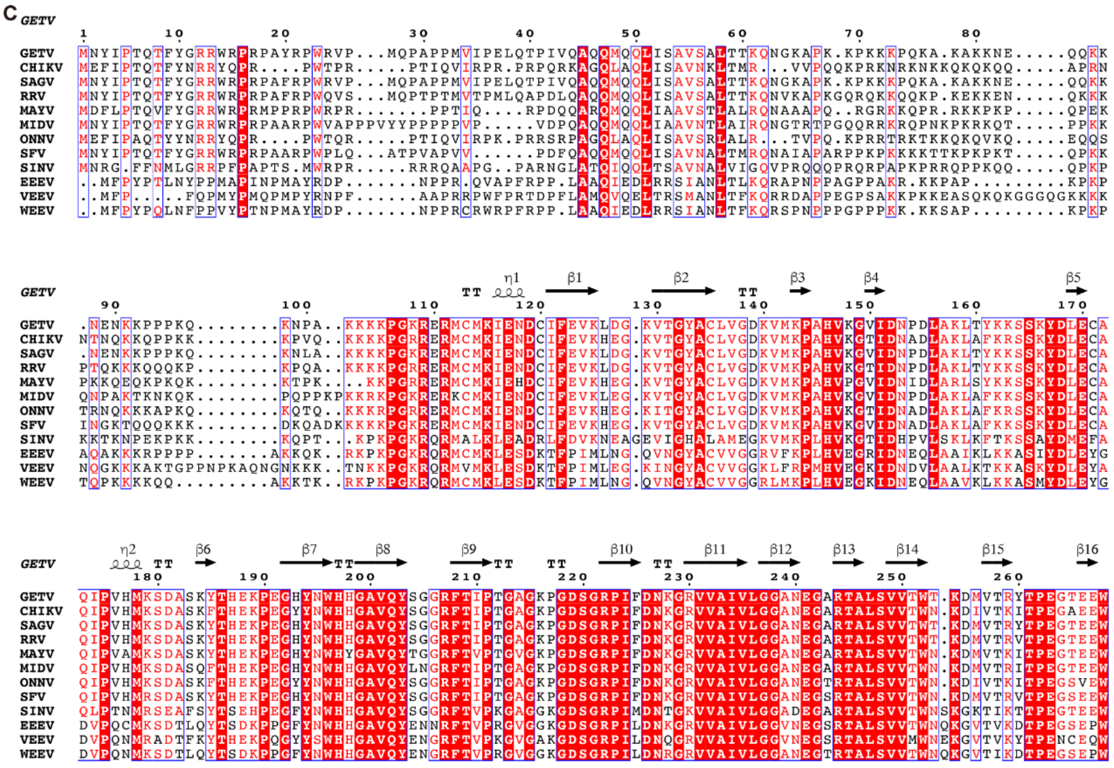

Figure S2. Multiple sequence alignment of the structural proteins from 12 alphaviruses. The alignment was done using the ClustalW and displayed with ESPript. The red background highlights the strictly conservative residues. The secondary structure determined by the Cryo-EM atomic structure model of the GETV is shown with linear diagram. (**A**) Alignment of E1. (**B**) Alignment of E2. (**C**) Alignment of CP.
