# Supplementary material for: Structural Insights into Alphavirus Assembly Revealed by the Cryo-EM Structure of Getah Virus
Source: Viruses. 2022 Feb 5;14(2):327. doi: 10.3390/v14020327 (PMC8876998; doi:10.3390/v14020327)
Supplement: Supplementary file 1 [file viruses-14-00327-s001.zip › Supplementary/supplementary Tables.pdf]

Table S1 Cryo-EM data collection and processing

Refinement	Envelope glycoprotein (EMD-32412)	Capsid protein (EMD-32426)
Initial model used (PDB code)	7WC2	7WCO
R.m.s. deviations		
Bond lengths (Å)	0.007	0.008
Bond angles (°)	1.270	1.187
Validation		
Clashscore	4.30	2.76
Poor rotamers (%)	0.62	0.53
Ramachandran plot		
Favored (%)	86.42%	85.84%
Allowed (%)	13.32%	14.16%
Disallowed (%)	0.26%	0.00%

GETV	Block-based reconstruction	
Protein	Envelope glycoprotein	Capsid protein
Electron Microscopy Data Bank	EMD-32412	EMD-32426
Magnification	18000X	
Voltage (kV)	300	
Electron exposure (e-Å-2)	30	
Nominal defocus (μm）	1~2.5	
Pixel size (Å)	1.36	
Symmetry imposed	icosahedron	
Number of micrographs	2555	
Initial number of particles images	36066	
Final number of particle images	30996	
Map resolution (Å)	3.5	3.8
FSC threshold	0.143	
Map resolution range (Å)	3.9-7.0	

Table S2 Amino acids involved in E2/E1 interface (intra-dimer) (d< 4Å)

E2		E1	
**Location**	**Residue**	**Residue**	**Location**
Domain A	Y18	T228	DII
Domain A	Y29	F87*, M88*, W89	DII
Domain A	R38	D112*	DII
Domain A	E40	V113	DII
Domain A	G72	W89	DII
Domain A	H73	W89	DII
Domain A	R138	D253*	DII
Domain A	T154	K181	DII
Domain A	E165	D112	DII
Domain B	P173	Y93	DII
Domain B	D174	M88, Y93*	DII
Domain B	I175	M88	DII
Domain B	P176	M88, G90, A92, Y93, F95	
Domain B	D177	G90	DII
Domain B	H226	F95*	DII
Domain B	A227	F95	DII
Domain B	A228	F95	DII
Domain A	T238	S57	DII
Domain A	S239	P56, S57*	DII
Domain A	S240	P58, V229, H230, V231	DII
Domain A	F241	V229, H230	DII
Domain A	V242	S57, P58, M88	DII
Domain A	P243	P58, I60, M88, Y93, V229,	DII
Domain A	R244	S57, P58*, Y59	DII
Domain A	A245	Y59	DII
Domain A	D246	Y59	DII
Domain A	Q247	Y59, R69, L103	DII
Domain A	L260	V113, H116	DII
Domain C	Y277	H386, I387	DIII
Domain C	G278	H386, I387	DIII
Domain C	K279	H386	DIII
Domain C	E281	I387	DIII
Domain C	T295	D253	DII
Domain C	R297	N252*, D253, K254, A255*, G258, C259*	DII
Domain C	S298	P256	DII
Domain C	L299	P256, F257, G258	DII
Domain C	G300	P256, F257	DII
Domain C	A301	F257	DII
Domain C	P303	K254, A255, P256	DII
Domain C	P305	K254	DII

Table S2 Amino acids involved in E2/E1 interface (intra-dimer) (d< 4Å) (Continued)

**E2**		**E1**	
**Location**	**Residue**	**Residue**	**Location**
Domain C	E307	T249	DII
Domain C	V320	I387	DIII
Domain C	E322	N396	DIII
Domain C	R336	G258*, V260, P389	DII, DIII
Domain C	L337	I387, V388, P389	DIII
Domain C	W338	H386, I387, V388*, P389, Y390, G391	DIII
Domain C	A339	H386, I387, V388	DIII
Domain C	Q340	S309, S310*, P383, D385, H386*, V388	DIII
Domain C	L341	Y59, P383	DII, DIII
Domain D	T342	P383*, D385, H386	DIII
Domain D	T343	P382	DIII
Domain D	K346	E381*	DIII
Domain D	P347	P382	DIII
Domain D	H348	A361, S362*, A379, C380, T405	DIII
Domain D	P351	W409	TM
Domain D	I354	A406	DIII
Domain D	Y357	H308*, P382	DIII
Domain D	Y358	A361, P400	DIII
Domain D	Y362	V398	DIII
TM	V377	L417	TM
TM	S380	L417, T421*	TM
TM	L381	L420	TM
TM	S384	L420, T421, A424	TM
TM	M387	A424, V425, L428	TM
TM	F388	A424	TM
TM	T390	L428	TM
TM	A391	V431	TM
TM	K394	V431, T432, T435*	TM
TM	C395	R438*	TM
TM	P398	R438	TM
TM	Y399	R438*	TM

* Residues involved in the formation of hydrogen bond
The hydrogen bonds were calculated using PISA (https://www.ebi.ac.uk)

Table S3 Amino acids involved in E1/E1 interfaces

Amino acids involved in E1/E1 interface i (d< 4Å)

E1		E1	
**Location**	**Residue**	**Residue**	**Location**
DII	T41	H125	DII
DII	K123	E151	DI
DII	H125	H125, T126	DII
DII	T126	H125	DII
DI	F147	R206	DII
DI	E151	P191, Y192	DII
DI	H152	R206	DII
DII	P191	E151	DI
DII	Y192	H152, T153	DI
DII	R206	F147, H152	DI

Amino acids involved in E1/E1 interface ii (d< 4Å)

E1		E1	
**Location**	**Residue**	**Residue**	**Location**
DI	F147	R206	DII
DI	N149	E45, K123	DII
DI	G150	P191	DII
DI	E151	K123, K176, P191, Y192	DII
DI	H152	P191, Y192, R206	DII
DI	T153	P191, Y192, G193, S194	DII
DI	V154	Y192, G193	DII
DI	T155	Y214	DII
DI	R160	G193, S194, G195, Q196, Y214	DII

Amino acids involved in E1/E1 interface iii (d< 4Å)

E1		E1	
**Location**	**Residue**	**Residue**	**Location**
DIII	V305	G23	DI
DIII	E381	G22	DI

Table S3 Amino acids involved in E1/E1 interfaces (continued)

Amino acids involved in E1/E1 interface iv (d< 4Å)

E1		E1	
**Location**	**Residue**	**Residue**	**Location**
DIII	V305	G22, V290	DI
DIII	E381	G22	DI

Amino acids involved in E1/E1 interface v (d< 4Å)

E1		E1	
**Location**	**Residue**	**Residue**	**Location**
DI	N22	P382, P383, K384, D385	DIII
DI	I291	D311	DIII
DIII	A295	I315	DIII
DIII	T297	V303, A304	DIII
DIII	N298	Q302	DIII
DIII	D323	T353	DIII

Table S4 Amino acids involved in E2/E2 interface (d< 4Å)

α/β		β/γ		γ/α		α′/β′#	
α	β	β	γ	γ	α	α′	β′
A92	G23	Y18	R266	A92	G23, D24	A20	R144, P145, H146
R94	D24, Q26	A20	R144	R94	D24, Q26	D21	R144, P145, H146
Y104	D24	D24	R94, Y104	Y104	D24, G25	G23	A92
T142	Q128	G25	R144	T142	Q128	D24	R94, Y104
V143	K126, Q128	F27	R144	V143	E110, K126, Q128	G25	Y104, R144
R144	A20, D21, G25, F27	D109	T142	R144	A20, G25 F27	Q26	R94
		K126	V143			D109	T142
		Q128	V143, H290			E110	V143
						K126	V143
						V127	V143
						Q128	H290
						K130	D289

The interactions of E2 at i3 are identical

Table S5 Glycans interaction with E1 and E2 (d< 4Å)

Glycan linked residue	Residue from E1 and E2
E1-N141	E1-R134
E2-N200	E1-C63, E1-C96, E1-E99, E2-T202
E2-N262	E1-H116, E1-N264, E2-L260, E2-T261
